# The Nashville Meeting

**Published:** 1870-09

**Authors:** 


### EDITORIAL DEPARTMENT.

*The Nashville Meeting.*—The tenth annual meeting of the American Dental Association held last month in Nashville, Tenn. the proceedings of which we present to our readers in the present number of the Journal, was a very interesting one, the majority of the members of the profession present being from the South and West.

Dr. W. H. Morgan of Nashville, whose name is a familiar one throughout the South, was elected President, a position he was justly entitled to years ago, and with which he has now been honored by the votes of those who appreciate his labors in behalf of Dentistry, without regarding political opinion and residence objectionable features, as has been the case heretofore.

The following letter exposes some sharp practice at the close of the meeting, similar to that in which the delegates to the political coventions of our country are wont to indulge in. It is scarcely necessary to remark that the Southern members were not the operators in the "wire working."

*Nashville, Tenn. August* 15*th* 1870.

Editor American Journal of Dental Science :

Dear Sir : For fear every body don't know that we are interested in dentistry down here, I send a few lines for your Journal, hoping they will inform every one that we are. If every one had attended the dental meetings, and heard the dental speeches, and dental talk, and dental manœuvreings we have had here, and here about lately, they would certainly think we had dentistry on the brain.

Our Tennessee Association met at Murfreesboro, about thirty miles from this place, on the 28th of July. We had a most excellent meeting—one of the most interesting we ever had.

This city (Nashville) was chosen as the next place of meeting, also as officers, Drs, C. P. Baird, President ; H. M. Acsee and A. Hartman, Vice Presidents ; S. H. Beard, Corresponding Secretary; R. Freeman, Recording Secretary, and Treasurer. The next meeting will commence on the fourth Thursday in July, 1871.

Now comes the American Dental Association, about one one hundred and thirty strong, to hold its tenth annual meeting, the first ever held south of Mason and Dickson's line, in the Representative Hall of our Capitol, a most excellent place for the occasion. Commencing August the 2nd at 10 A. M., continuing its sessions two a day, for four days, during which time we had a good deal of very pleasant and interesting discussion, as well as many other things done and said, calculated as we thought and and hoped to beget a very pleasant state of affairs betwixt the two sections of country. We began to feel that our labors for the past three or four years had not been in vain, that another meeting or two in the heart of the South would beget such a feeling and interest that nothing would shake it off, especially after we had selected, when all were present, Atlanta, Ga. for the next place of meeting. But unfortunately some few things occurred that we fear will spoil the whole. Just before adjourning, when a good many had gone home, and many others who had not were absent, a motion was made and pushed through, for the purpose of reconsidering the vote making Atlanta the next place of meeting, and fixing Niagara Falls instead.

After this very ungenerous as well as unfair thing had been done, and things began to look a little stormy, some of the better thinkers concluded another reconsideration would be in order, and such a motion was made and carried, resulting in a move from Niagara to White Sulphur Springs, Va. The compromise we fear will not have that effect that many seem to think. To say the least, we have our doubts about reinstating the feeling that existed before the motion was made to remove from Atlanta.

We have labored, as many others have done, for several years, to make this society truly national, but we must confess at present we feel that all has been done in vain, we hope, however, that our feelings may not be realized. While we trust that something else may be done to interest the South in this society, we are satisfied that our first as well as most golden opportunity has been lost. Atlanta would have given us fully one hundred representative men of the South, to have joined shoulder to shoulder with with the North in trying the great national Dental wheel of science at the next meeting. As it is we fear, the present place in the South, as it may be called, will not add one fourth or fifth that number. The whole thing is chargeable to a few individuals."

*Dr, W. W. H. Thackston.*—We are pleased to learn that this gentleman, one of the most eminent practitioners of dentistry in the South, has been elected Mayor of Farmville, Virginia, where he has long resided. Dr. Thackston is one of the early graduates of the Baltimore College of Dental Surgery, of the class of 1842.

